# Primary Care Approach to Endometriosis: Diagnostic Challenges and Management Strategies—A Narrative Review

**DOI:** 10.3390/jcm14134757

**Published:** 2025-07-04

**Authors:** Marta Ortega-Gutiérrez, Antonio Muñoz-Gamez, María de la Sierra Girón-Prieto

**Affiliations:** 1 Faculty of Medicine, University of Granada, 18016 Granada, Spain; martaogz@correo.ugr.es; 2 Centro de Salud de Armilla, 18100 Granada, Spain; antoniomunozgamez@gmail.com

**Keywords:** endometriosis, primary health care, diagnosis, risk factor, ultrasound, treatment

## Abstract

Endometriosis is a chronic inflammatory disease characterized by the presence of ectopic endometrial tissue, mainly in the pelvic cavity. It primarily affects women of reproductive age and is associated with significant morbidity, particularly chronic pelvic pain and infertility. Despite its high prevalence, diagnosis is often delayed, contributing to prolonged suffering and increased healthcare burden. This review examines the management of endometriosis in Primary Care, focusing on clinical presentation, risk factors, diagnostic approaches, and therapeutic options. A comprehensive bibliographic search was conducted using PubMed, Scopus, and Uptodate, including evidence-based clinical guidelines and literature up to January 2025. Women diagnosed with endometriosis in Primary Care are typically of reproductive age, with symptoms including dysmenorrhea, dyspareunia, and abnormal uterine bleeding. Risk factors include early menarche, low birth weight, short menstrual cycles, and family history. Transvaginal ultrasound is the recommended first-line imaging tool. Treatment includes analgesics, nonsteroidal anti-inflammatory drugs (NSAIDs), and hormonal therapies such as combined oral contraceptives or progestins. Non-pharmacological interventions, including dietary modifications and psychological support, are also relevant. Early identification in Primary Care is key to improving out-comes. Enhancing awareness among healthcare providers and promoting multidisciplinary management are essential to optimize care and reduce diagnostic delays.

## 1. Introduction

**Figure 1:**
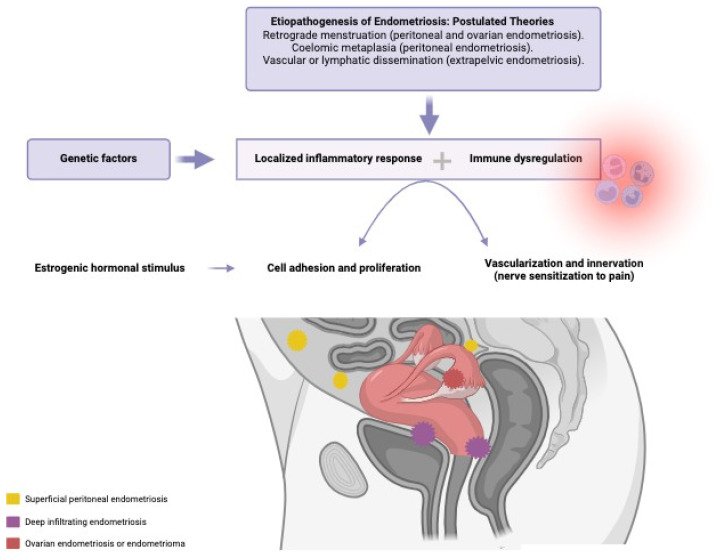
Etiopathogenesis of endometriosis, interaction of the involved factors. Created in Biorender.com.

Endometriosis is defined as the presence of ectopic endometrial tissue, mainly in pelvic organs and tissues [1]. It is a complex disease both symptomatically and etiopathogenically, with an etiology that remains imprecise. The most widely accepted theories include retrograde menstruation, coelomic metaplasia, and vascular or lymphatic dissemination [2,3]. The pathogenesis of this estrogen-dependent inflammatory disease involves endocrine, immunological, proinflammatory, and proangiogenic mechanisms. A localized immune and inflammatory response occurs, with the production of cytokines, chemokines, and prostaglandins [2]. Thus, the etiology appears to be multifactorial, see Figure 1, including the transport of ectopic endometrial tissue from the uterus to the peritoneal cavity, along with a conducive peritoneal environment, characterized by altered immunity, imbalanced cell proliferation and apoptosis, abnormal endocrine signals, and genetic factors [4]. There are different types of endometriosis based on its location: superficial peritoneal endometriosis, ovarian endometrioma, and deep infiltrating endometriosis. This classification is important, because symptoms vary depending on lesion location, and responses to hormonal treatment may differ accordingly [5]

### 1.1. Rationale

Endometriosis is one of the most common gynecological disorders worldwide [6]. It is estimated to affect between 3% and 10% of women of reproductive age [2,4] and up to 50% of infertile patients or those undergoing diagnostic laparoscopy for pelvic pain [7]. Despite its high prevalence, the disease is often underrecognized in clinical practice, leading to frequent misdiagnoses and suboptimal care [7].

The condition causes substantial morbidity, particularly due to pain and infertility [7]. Chronic or severe pain symptoms significantly impair quality of life [6,8]. Pelvic pain, dysmenorrhea, dyspareunia, dysuria, dyschezia, fatigue, and infertility not only affect physical well-being, but also mental, sexual, and social health [2]. As a public health issue, endometriosis entails considerable individual and societal costs [9]. Women diagnosed with endometriosis face a significantly higher burden of infertility and chronic comorbidities [10], as well as increased healthcare utilization and associated costs—particularly among younger patients, whose healthcare needs may differ from those of healthy women [11].

Although endometriosis tends to progress, its natural course remains uncertain and difficult to predict due to the unclear pathogenesis [6,12]. Early detection is thus critical to prevent advanced pain syndromes and reproductive complications resulting from disease progression [6].

Diagnostic delay represents a major issue for women with endometriosis [6]. On average, patients experience a delay of 7 to 10 years between symptom onset and diagnosis [7,13]. During this time, they endure significant symptoms, without adequate treatment, often requiring more complex interventions than would have been necessary with early detection. Furthermore, treatment efficacy may decrease as the disease progresses [6].

Therefore, early diagnosis is vital to prevent pain and disease advancement. Awareness and knowledge of endometriosis among healthcare professionals are key [6]. In this context, Primary Care plays a crucial role. Women with endometriosis often consult their general practitioners, presenting opportunities for earlier diagnosis [8]. Primary Care providers are well positioned to mitigate diagnostic delays by maintaining a high index of suspicion, recognizing common presentations, and facilitating timely referrals [7,14]. Once diagnosed, continued support from Primary Care professionals is essential, due to the chronic nature of the disease. As patients’ needs and priorities evolve over time, treatment strategies must be adjusted accordingly [6].

Given its high prevalence and significant impact on women’s quality of life, optimizing endometriosis management at the Primary Care level is imperative. This setting represents the initial point of contact with the healthcare system, making it an ideal platform for early recognition and intervention. For this reason, synthesizing current knowledge on endometriosis management in Primary Care is the objective of this review.

### 1.2. Objectives

The objective of this literature review is to compile information regarding the management of endometriosis in the Primary Care setting. Specifically, it aims to analyze the key signs and symptoms that may support early diagnosis, as well as associated risk factors. Additionally, this review evaluates the role of ultrasound in diagnosis and outlines the therapeutic options available for treating endometriosis in Primary Care.

## 2. Materials and Methods

This review seeks to address the following research questions:

- What are the clinical characteristics of patients diagnosed with endometriosis in Primary Care?
- What risk factors are associated with endometriosis?
- What are the main signs and symptoms suggestive of endometriosis in Primary Care?
- What is the role of ultrasound in the diagnosis of endometriosis in this context?
- What treatment options are available in Primary Care for managing endometriosis?

A narrative review was conducted focusing on the management of endometriosis in the Primary Care setting. The search strategy included the use of the PubMed, Scopus, and UpToDate databases, covering studies published up to January 2025. The search equation used was as follows: **((endometriosis and diagnosis)) OR ((endometriosis and risk factor)) OR ((endometriosis and ultrasound)) OR ((endometriosis and treatment)) AND ((primaryhealth care)).**

The search was limited to the following: (a) Publications related to patients diagnosed with endometriosis, regardless of severity or clinical presentation, whose management took place in Primary Care. (b) Any type of epidemiological study (clinical trials, cohort studies, case-control studies, and case series). (c) Articles written in Spanish or English. In contrast, the following were excluded: (a) Publications that do not address the management of endometriosis in the context of Primary Care. (b) Protocols and conference abstracts. (c) Articles written in a language other than Spanish or English.

An initial search was conducted to screen the titles and abstracts of all studies retrieved using the search equation across the databases. The full texts of all studies meeting the inclusion and exclusion criteria were then reviewed, along with their reference lists to identify additional sources. Articles with unclear eligibility were discussed and resolved in consensus with a second reviewer. Studies deemed relevant were included in the review. Eligible sources included epidemiological studies, relevant prior reviews, and evidence-based clinical guidelines.

## 3. Results

Following the bibliographic search conducted in PubMed, Scopus, and UpToDate, a total of 31 articles were selected after removing duplicates and applying predefined selection criteria. An additional 16 sources were retrieved through manual review of the reference lists of these articles. Furthermore, this review included data from the most recent version (November 2024) of the NICE guidelines (National Institute for Health and Care Excellence), which is based 106 on the best available current evidence.

### 3.1. What Is the Typical Profile of Women Diagnosed with Endometriosis in Primary Care?

Understanding the demographic and clinical characteristics of women diagnosed with endometriosis in Primary Care helps can identify pattern indicative of the disease in this setting.

The prevalence of endometriosis is approximately 10% among women of reproductive age [2,4]. However, estimating its prevalence in the general population is challenging, due to the presence of asymptomatic cases and the variability and nonspecificity of symptoms in symptomatic patients [4]. Demographically, these are typically women of reproductive age, as the disease is estrogen-dependent [6]. Symptoms may begin during adolescence and usually stabilize after menopause [7]. Peak prevalence occurs between the ages of 25 and 35 [15], although it can develop at any point during reproductive life [16]. A 2022 study by Medina-Perucha et al. reported lower endometriosis prevalence in women from low-income and rural areas, possibly due to barriers to access to diagnosis. More research is needed to understand how social inequalities influence endometriosis diagnosis and women’s health outcomes [17].

Clinically, the presentation is highly variable [6,16]. Patients may range from asymptomatic to exhibiting typical symptoms such as debilitating pelvic pain and infertility [6,18]. In addition to gynecological symptoms, systemic manifestations may occur [16]. Common comorbidities include migraine or headache, upper respiratory tract infections, allergic rhinitis, and contact dermatitis or eczema. Mental health conditions, particularly anxiety, are also frequently associated [16].

In summary, the typical patient profile in Primary Care includes women of reproductive age, potentially even adolescents. Special attention should be paid to women from lower socioeconomic backgrounds or rural areas, who may be at higher risk for delayed diagnosis.

Clinically, these patients often present with classic symptoms, particularly pelvic pain, and those with fertility concerns frequently seek medical advice, as infertility is more prevalent in this group. Many also experience comorbid conditions that further reduce quality of life. Due to diagnostic delays and associated comorbidities, these patients tend to utilize healthcare services more frequently. Studies have shown that women with endometriosis use both primary and secondary care more extensively in the ten years prior to diagnosis compared to women without endometriosis [11,19]. These findings underscore the importance of early disease detection in Primary Care (Table 1).

### 3.2. What Risk Factors Are Associated with Endometriosis?

Endometriosis is influenced by both genetic and environmental factors [2,20]. A strong genetic component has been identified, with increased disease prevalence among first-degree relatives of affected individuals [2,20,21,22]. Family history is therefore a well-established risk factor. Twin studies estimated the heritability of endometriosis to be approximately 50% [20,21].

Beyond genetic predisposition, environmental exposures—especially during intrauterine and early life stages—are believed to play a significant role by affecting gene expression [20,21]. Critical periods of exposure, including fetal development and early childhood, are thought to influence later disease onset [2,20].

Among intrauterine exposures, diethylstilbestrol [2,4,20,23]—a synthetic estrogen formerly used to prevent miscarriage—is a noteworthy risk factor. Maternal smoking during pregnancy has also been implicated [23]. In early childhood, low birth weight (under 2.5 kg) and prematurity are recognized as significant risk factors [2,20,23]. Other proposed influences include passive smoking and formula feeding during infancy, compared to breastfeeding [23].

In adolescence, early menarche (before ages 11–13) [4], low body mass index (BMI) [2,4,20], and low waist-to-hip ratio [2] are all associated with increased risk of endometriosis. In adulthood, prolonged exposure to endogenous estrogens is particularly relevant [4]. This includes short menstrual cycles (fewer than 27 days) [4], long or heavy periods, and low parity or nulliparity [2,4,20] (Table 2).

While these associations are well documented, it remains unclear whether they represent true causative factors or are consequences of the disease itself. The pathogenesis of endometriosis is not yet fully understood [2,24]. More research is needed to clarify its etiological mechanisms and further identify and validate associated risk factors and their biological foundations [23].

### 3.3. Diagnostic Management: What Are the Signs and Symptoms Suggestive of Endometriosis in Primary Care?

Diagnosing endometriosis presents a significant clinical challenge, and diagnostic delay remains one of the most critical issues associated with the disease. Most adult women with endometriosis report the onset of symptoms during adolescence; however, early treatment is rare [2]. On average, diagnostic delays span 4 to 10 years [7,13,20], with patients typically consulting up to seven different healthcare providers before receiving a definitive diagnosis [2].

This prolonged delay can lead to disease progression, increased pain, psychological distress, infertility, and reduced quality of life [2,20].

Diagnostic difficulties arise from both the nature of the disease and the approach taken by healthcare providers and patients [13,18]. Endometriosis often presents with nonspecific symptoms that overlap with other benign conditions and lacks reliable biomarkers [2,7,25]. In addition, cultural factors—such as stigma or the normalization of symptoms—may prevent patients from seeking help. A key modifiable barrier is the lack of awareness and low clinical suspicion among healthcare professionals [2,7].

Thus, raising awareness and improving knowledge about endometriosis among health-care providers is essential for early diagnosis [6]. Primary Care practitioners can play a pivotal role in this regard, helping patients recognize that their symptoms are not normal and increasing diagnostic suspicion based on thorough clinical histories [20,25]. This awareness can be enhanced through educational interventions, diagnostic support tools, and adapted clinical guidelines [26].

#### 3.3.1. Symptoms Most Commonly Associated

The clinical presentation of endometriosis is highly variable and heterogeneous [2,14]. Although some patients remain asymptomatic, the majority experience symptoms such as chronic pelvic pain and infertility [5,6,14].

The most frequently reported gynecological symptoms include

- Chronic pelvic pain (cyclical or continuous for more than six months, affecting 37%).
- Dysmenorrhea (often more severe than primary dysmenorrhea, 62%).
- Abnormal uterine bleeding (heavy and/or prolonged menstrual flow, 51%) [6,20].

Additional symptoms vary depending on the location of endometrial implants. Deep infiltrating endometriosis may lead to

- Dyspareunia (pain during or after penetrative intercourse),
- Dyschezia (painful defecation),
- Gastrointestinal or urinary symptoms, such as altered bowel movements, cyclic rectal bleeding, rectal pain radiating to the perineum, hematuria, or dysuria (Bianchi_intestinal_nodate [2,6,14,15,27]).

Thoracic or abdominal wall endometriosis—though rare—may present with umbilical masses that bleed during menstruation [14,28]. Notably, symptom severity does not always correlate with disease stage [6,14].

Due to the estrogen-dependent nature of endometriosis, symptoms often worsen during menstruation and improve during pregnancy or after menopause [14]. Any symptom that consistently worsens with the menstrual cycle should prompt consideration of endometriosis [7], although symptoms may also occur independently of the cycle due to potential central sensitization and a neuropathic component [2,6,29].

In Primary Care, endometriosis should be suspected in women of reproductive age who report severe dysmenorrhea, pelvic pain, and abnormal uterine bleeding—especially if accompanied by dyspareunia, dyschezia, or urinary or gastrointestinal symptoms. The cyclic pattern of symptom exacerbation during menstruation is particularly characteristic.

#### 3.3.2. Medical History and Physical Examination

Taking a history is essential to guide the diagnosis of endometriosis in primary care [14] (Table 3). A detailed clinical interview should include questions about

- Dysmenorrhea, dyspareunia, chronic pelvic pain.
- Cyclical gastrointestinal or urinary symptoms.
- The relationship of symptoms to the menstrual cycle.
- Pain intensity, response to analgesics, and associated symptoms [7,14].

Equally important is assessing the impact of symptoms on quality of life, mental health, and daily activities [14]. Disease severity does not always correlate with the extent of anatomical involvement [7]. Fertility desires must also be explored, as these will influence treatment decisions [14]. For patients who have had children, reproductive history and any difficulties conceiving should be documented [7].

Other relevant aspects to investigate include

- Family history of endometriosis.
- Intrauterine and early-life risk factors (e.g., low birth weight, early menarche).
- Associated comorbidities [12].

The physical examination, following a suggestive clinical history, should include a complete abdominal and pelvic evaluation, including speculum and bimanual examination, and transvaginal ultrasound when indicated and with the patient’s consent [14,30,31].

While history-taking has high sensitivity but low specificity, physical examination contributes additional diagnostic value through high specificity and a high positive likelihood ratio. Therefore, in Primary Care, pelvic examination enhances diagnostic accuracy when combined with clinical history [31].

Findings may include

- Pelvic organ fixation.
- Palpable endometriotic nodules or adnexal masses.
- Visible vaginal lesions, tenderness in the posterior fornix [6,7,30,31].

Pain elicited on palpation is a typical feature [31]. However, examination findings may be subtle [6], and the absence of abnormal findings does not rule out a diagnosis [6,7,14,31].

In conclusion, a positive physical examination increases the diagnostic value when combined with a thorough history. Educating patients on the role and value of examination fosters trust and facilitates empathetic care [31].

#### 3.3.3. Complementary Laboratory Tests

A non-invasive diagnostic test could potentially allow for faster and easier diagnosis of endometriosis. Numerous biomarkers have been proposed, including CA-125, HE-4, and PGP 9.5. If proven to be sufficiently accurate, a blood test could become a safe and cost-effective diagnostic tool accessible in Primary Care settings [6]. However, to date, laboratory diagnosis of endometriosis remains a challenge.

CA-125 levels may be elevated in women with endometriosis, but due to its low sensitivity and specificity, it is not recommended as a standalone diagnostic marker [6,14,20,32,33]. Nevertheless, if CA-125 levels are available incidentally, a serum concentration > 35 IU/mL may support a diagnosis—although normal levels do not exclude it [6,33].

Other biomarkers, such as HE-4 (commonly used in ovarian cancer) and PGP 9.5 (linked to nerve fibers in endometrial tissue), have also failed to demonstrate adequate diagnostic performance. PGP 9.5, in particular, lacks specificity, is not validated, and is expensive [6,14].

#### 3.3.4. Confirmatory Diagnosis and Staging Systems

The gold standard for confirming endometriosis is laparoscopy with biopsy, which allows for histological confirmation of suspicious lesions [20]. However, due to its invasiveness and limited cost-effectiveness, diagnostic laparoscopy is no longer considered necessary to initiate treatment [14,34]. Advances in imaging techniques now support a predominantly clinical diagnosis, helping to avoid treatment delays [20,35].

Several classification systems are used to stage endometriosis, typically based on anatomical location, extent, and depth of invasion. A widely adopted system is the revised American Society for Reproductive Medicine (rASRM) classification, which categorizes the disease into four stages [6].

However, staging systems do not reliably correlate with symptom severity or clinical impact. Some women with minimal disease may experience severe symptoms, while others with extensive disease may be relatively asymptomatic. Therefore, treatment strategies should prioritize individual symptoms, preferences, and goals, rather than relying solely on staging [6].

#### 3.3.5. Referral Criteria from Primary Care

The management of endometriosis in Primary Care should be coordinated with specialized gynecology services. Clearly defined referral criteria are necessary to ensure that patients receive the most appropriate care for their needs [6].

Referral to general gynecology services for further investigation and management should be considered in the following cases:

- First-line treatment is ineffective, poorly tolerated, or contraindicated.
- Symptoms interfere with daily activities.
- Symptoms are persistent or recurrent.
- Pelvic signs of endometriosis are present, but deep infiltrating endometriosis is not suspected.

If fertility is a current priority at the time of diagnosis, referral is also essential, as management of endometriosis-related subfertility requires a multidisciplinary team, including fertility specialists [6].

Referral to a specialized endometriosis center is warranted in cases of suspected or confirmed

- Endometrioma.
- Deep infiltrating endometriosis (e.g., involving bowel, bladder, or ureters).
- Extrapelvic endometriosis.

Additionally, suspected or confirmed adolescent cases should be referred to adolescent gynecology or endometriosis specialist services for comprehensive assessment and management [6].

### 3.4. What Is the Role of Ultrasound in the Diagnosis of Endometriosis in Primary Care?

Transvaginal ultrasound is currently considered the first-line imaging modality for diagnosing endometriosis [12,14,15,20,36]. It serves as an intermediate step between empirical treatment—based solely on symptoms—and diagnostic laparoscopy [6,37]. This preference is due to its accessibility, low cost, and high sensitivity and specificity [14,15,20].

The diagnostic accuracy of ultrasound depends on the presentation of the disease and the expertise of the operator [6,15]. Consequently, a negative ultrasound does not exclude endometriosis. If clinical suspicion remains high despite a normal result, a referral to a more specialized imaging service should be considered [6,38].

Endometriomas can be reliably identified via transvaginal ultrasound, with sensitivity and specificity rates exceeding 90% [2,6,37]. The use of Doppler ultrasound can further improve diagnostic precision in ovarian endometriosis [14]. Experienced sonographers may also detect deep infiltrating endometriosis and pelvic organ adhesions with transvaginal imaging [2,6,37,38]. However, the sensitivity and specificity of imaging techniques are limited when it comes to detecting superficial peritoneal lesions [2,6,14].

The IDEA (International Deep Endometriosis Analysis) Group developed a standardized transvaginal ultrasound protocol for endometriosis diagnosis. This includes four main components: assessment of the uterus and adnexa, search for deinfiltrating endometriosis, evaluation of organ sliding, and identification of soft ultrasound markers [36]. Dynamic ultrasound enables assessment of both static findings (e.g., endometriomas, hydrosalpinx, hypoechoic nodules) and functional aspects (e.g., obliteration of the pouch of Douglas, organ mobility, and localized tenderness) [36] (Table 4).

MRI can be useful in determining the extent and precise location of endometriotic lesions [14]. While not a first-line imaging tool [6], MRI has a 94% sensitivity and 79% specificity for diagnosing deep endometriosis [2]. Like ultrasound, however, it is limited in detecting superficial peritoneal implants [2,14,37].

Therefore, transvaginal ultrasound (or abdominal ultrasound when vaginal access is not feasible or declined) should be offered in Primary Care to all women with suspected endometriosis, even if pelvic examination is normal [6,12]. The aim is to identify ovarian endometriomas and deep infiltrating lesions, including those affecting the bowel, bladder, or ureters, as well as to rule out alternative causes of symptoms. Ultrasound findings will inform clinical management and referral decisions [6,36].

Importantly, a normal ultrasound or MRI should not exclude the diagnosis of endometriosis. If clinical suspicion and symptoms persist, referral to a specialized unit for further evaluation is recommended to avoid perpetuating diagnostic delays [6].

### 3.5. What Therapeutic Options Are Available for the Treatment of Endometriosis in Primary Care?

Healthcare professionals must recognize that endometriosis is a chronic condition with complex and evolving needs that require long-term, patient-centered support [6,39]. Management strategies should be tailored to the patient’s life stage, predominant symptoms, personal preferences, reproductive goals, and the impact of the disease on daily functioning and overall well-being [6,39].

**Figure 2:**
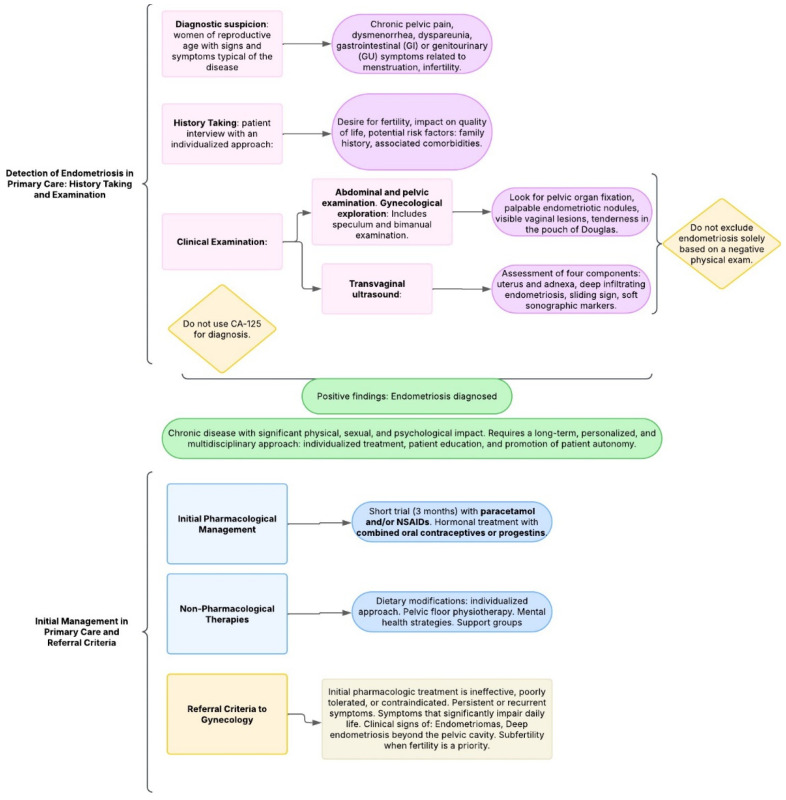
Proposed diagram: management of endometriosis in primary care.

Providing comprehensive information to patients with suspected or confirmed endometriosis is essential. This includes clear explanations of the condition, available treatment options, and supportive resources [6]. Empowering patients to take an active role in managing their symptoms enhances both treatment outcomes and patient satisfaction. Thus, effective care for endometriosis should follow a personalized and multidisciplinary approach [39]. The following is a proposed diagram for the detection and management of endometriosis in Primary Care (Figure 2).

#### 3.5.1. Medical Treatment

Before initiating pharmacological treatment, clinicians should discuss the benefits, risks, and potential side effects of medications. Individual comorbidities and the patient’s goals regarding pain control and fertility must also be considered [6,39,40].

A key component of medical treatment is pain management, as pelvic pain is the most disabling and prevalent symptom. This pain often includes an inflammatory and neuropathic component, with potential for central sensitization, leading to chronic pelvic pain [2,6].

In Primary Care, first-line pharmacological management includes paracetamol (acetaminophen) and nonsteroidal anti-inflammatory drugs (NSAIDs), either separately or in combination [6,41]. If there is no adequate pain relief after a 3-month trial, referral to specialized care should be considered [6].

Hormonal therapy is aimed at suppressing estrogen, thereby reducing ectopic endometrial proliferation and associated inflammation [14,20,39,41]. Long-term hormonal treatment can also help prevent recurrence [6,14,39]. No single hormonal agent is specifically preferred, but combined oral contraceptives (COCs) or progestins administered in continuous regimens are considered first-line treatments in Primary Care due to their favorable efficacy and tolerability profiles [2,6,14,39,40].

If first-line hormonal therapy is contraindicated or poorly tolerated, patients should be referred to specialized services [6] to explore other therapies, such as

- Gonadotropin-releasing hormone (GnRH) agonists and antagonists, which are considered a second-line therapy, induce systemic hypoestrogenism but are associated with risks such as bone loss and cognitive or cardiovascular side effects [6,12,39,40,42].
- Aromatase inhibitors are considered third-line hormonal treatment, typically reserved for complex or refractory cases [6,12,40,43].

#### 3.5.2. Non-Pharmacological Therapies

Many women choose to incorporate non-pharmacological treatments, either as alternatives or complements to medical therapy. These strategies offer patients greater autonomy in managing their condition and are especially appealing to those avoiding hormonal treatments, such as women trying to conceive. However, robust evidence supporting these approaches is still lacking [6,39].

Emerging data suggest that pro-inflammatory diets may worsen endometriosis symptoms, whereas anti-inflammatory and gluten-free diets may alleviate them [39]. Individualized dietary adjustments have been associated with symptom improvement and enhanced well-being, although further research is needed to support evidence-based dietary recommendations [44].

In patients with persistent pelvic pain, pelvic floor dysfunction is common and pelvic floor physical therapy can provide relief from symptoms after appropriate evaluation [39].

Given the high prevalence of anxiety and depression in women with endometriosis, addressing mental health is critical. Psychological stress can exacerbate pain perception. Psychological care should be multidimensional [39,45], with cognitive behavioral therapy (CBT) being the most established approach. Additional interventions such as mindfulness-based therapies [39,46] and Acceptance and Commitment Therapy (ACT) show promise, though evidence remains limited [46].

Finally, support groups are valuable for patient empowerment and information exchange [6]. In Spain, the Asociación de Afectadas de Endometriosis Crónica Estatal (ADAEC) offers resources for affected women [47].

## 4. Discussion

Interpreting the findings within the context of existing literature, this review confirms that the clinical presentation of endometriosis is variable and heterogeneous [2,4], as consistently described in previous studies. The core symptoms—such as chronic pelvic pain, dysmenorrhea, dyspareunia, and infertility—are well-established and reaffirmed across the scientific literature.

As evidenced, in women with suspected endometriosis, a positive pelvic examination significantly enhances diagnostic accuracy when combined with a thorough clinical history and symptom assessment [32]. Furthermore, transvaginal ultrasound remains the imaging modality of choice for initial diagnosis in Primary Care due to its diagnostic utility, accessibility, and cost-effectiveness [12,14,15,20,36].

The therapeutic approach must be individualized, taking into account each patient’s clinical profile, personal priorities, and preferences [6]. Pain management is a cornerstone of treatment [2,6,40], and combined oral contraceptives or progestins are recommended as first-line hormonal therapy in Primary Care, due to their efficacy and tolerability [2,6,14,39,40].

This review also underscores the need for continued research in several areas:

- The role of social inequalities in diagnostic access [17].
- Further clarification of the etiopathogenesis and associated risk factors [23].
- The limited evidence base regarding non-pharmacological interventions [6,39].
- The development of a non-invasive diagnostic test, which remains an important but unmet goal [6].

A notable limitation of this review is the heterogeneous quality of the included studies, many of which are themselves literature reviews. The lack of a systematic methodology also limits the reproducibility and comprehensiveness of the findings. Nevertheless, strengths of this work include the search strategy across multiple databases and the integration of clinical guidelines and high-quality evidence from systematic reviews and descriptive studies.

Ultimately, this review reaffirms the complexity and burden of endometriosis, as well as the critical role of Primary Care in addressing diagnostic delays. As consistently reported in the literature, empowering Primary Care providers with knowledge and diagnostic tools is essential to improve early detection and optimize the long-term management of endometriosis [6,7,15,20,26].

## 5. Future Directions

In the context of endometriosis management in Primary Care, there is still much to be investigated. From a biopsychosocial perspective, it is important to explore the impact of social inequities on the diagnosis of the disease. Moreover, higher-quality evidence is needed regarding the effectiveness of non-pharmacological therapies. Additionally, clarifying the etiopathogenesis of endometriosis is essential, as it would help identify the most relevant risk factors. In this regard, the role of endocrine-disrupting chemicals in menstrual blood is currently under investigation.

A primary research priority is the identification of a biomarker with sufficient sensitivity and specificity for the non-invasive diagnosis of endometriosis. Early detection through a reliable screening test would represent a major advancement in the clinical management of this disease [48,49,50].

Currently, research is ongoing into several potential biomarkers derived from serum, plasma, urine, and tissue samples. These include a wide range of biomolecules:

- Apoptosis markers
- Immune and angiogenic markers
- Glycoproteins
- Neuropeptides
- Hormones
- Oxidative stress markers
- MicroRNAs (miRNAs), and others.

These biomarkers may serve as biological signatures to facilitate diagnosis of underlying disease [50]. Promising research avenues include proteomics, metabolomics, genomics, microbiome analysis, and particularly miRNA profiling [50], which has emerged as one of the most promising approaches for developing non-invasive diagnostic tools. However, further validation is required [15,48,50].

Despite these advancements, no current biomarker has demonstrated the necessary reliability to serve as a standard clinical screening test. Therefore, future research must continue to focus on discovering robust diagnostic tools that can be translated into clinical practice [15,48,50].

## 6. Conclusions

In conclusion, endometriosis is a complex disease whose diagnostic delay represents a major challenge in its clinical management. Within the Primary Care setting, awareness and knowledge of the condition among healthcare professionals are essential for achieving early diagnosis.

The typical profile of patients diagnosed at the Primary Care level corresponds to women of reproductive age, often presenting with characteristic symptoms of the disease and associated comorbidities that negatively impact quality of life. The most relevant risk factors include: a family history of endometriosis, intrauterine and early-life exposures (e.g., low birth weight), and prolonged endogenous estrogen exposure during adulthood.

The clinical presentation is highly variable, with the most common symptoms being chronic pelvic pain, dysmenorrhea, and abnormal menstrual bleeding, often accompanied by dyspareunia, dyschezia, hematuria, dysuria, or infertility. Ultrasound, particularly the transvaginal approach, plays a fundamental role in diagnosis and is currently the imaging test of choice in Primary Care, due to its value in guiding clinical decisions and referrals.

Treatment strategies should emphasize individualized, long-term care, promoting patient education and autonomy. Analgesics and hormonal therapy (oral contraceptives or continuous progestins) constitute the first-line pharmacologic approach. Additionally, non-pharmacological therapies—such as dietary interventions, pelvic floor physiotherapy, mental health support, and peer support groups—are valuable resources that empower patients in the self-management of endometriosis.

## Figures and Tables

**Table 1:** Summary of the profile of women diagnosed with endometriosis in Primary Care.

Demographic	Characteristics Clinical Characteristics
Women of reproductive age	Variable presentation. Cyclic pelvic pain and infertility.
	Medical comorbidities: headache, migraine, upper respiratory tract infections (URTI), allergic rhinitis, contact dermatitis.
	Mental health comorbidities: anxiety and depression.

**Table 2:** Risk factors associated with endometriosis according to the woman’s life stage.

Genetic	First-Degree Relative Diagnosed with Endometriosis
In utero/Early childhood	Exposure to diethylstilbestrol, maternal smoking, low birth weight, prematurity.
Childhood and adolescence	Early menarche, low body mass index, low waist-to-hip ratio.
Adulthood	Short menstrual cycles, prolonged menstrual bleeding, heavy menstrual flow, low parity, nulliparity.

**Table 3:** Interview proposal on endometriosis indicators to create a clinical history of patients suspected of having the disease.

Family History	Do you have a first-degree relative (mother, sister) who suffers from endometriosis?
Risk Factors in Uterus or Early Childhood	Do you know if you were born with low birth weight? Were you born prematurely?
Gynecological History Since Adolescence	At what age did you first get your period? Was it at an early age? Were your periods painful from adolescence? Has your daily life been affected by the pain associated with menstruation? Does the pain associated with menstruation respond poorly to common painkillers like NSAIDs?
Associated Symptoms	Are your periods very painful? Do you experience pain during sexual intercourse? Do you experience pelvic pain even when not menstruating? Do you have digestive symptoms, such as intestinal pain or pain when defecating? Do you have urological symptoms, such as discomfort when urinating?
Pain History	Do you notice that the pain is associated with your menstrual cycle? How intense is the pain? Is there any relief from the pain, such as any medication?
Associated Comorbidities	In addition to these symptoms, do you experience fatigue or anxiety? Are you of an atopic constitution? Do you suffer from migraines?
Infertility	Have you been a mother or do you know if you would like to be in the future? If you have been a mother, how were your pregnancies? Did you have difficulties conceiving?
Impact on Quality of Life	How do the symptoms impact your quality of life?

**Table 4:** Ultrasound evaluation components, objectives, and associated symptoms in endometriosis assessment.

Component Evaluated	Objective	Associated Symptoms
Uterus and Adnexa	Detection of adenomyosis, endometrioma, hydrosalpinx or hematosalpinx.	None or typical endometriosis symptoms: pelvic pain, dysmenorrhea, dyspareunia, infertility.
Deep Infiltrating Endometriosis (DIE)	Search for hypoechoic nodules in the anterior (from anterior uterine serosa to anterior pelvic wall) and posterior compartments (from posterior uterine serosa to presacral space).	Anterior Compartment (Bladder): urinary urgency, dysuria, hematuria. Posterior Compartment (Bowel, rectum and rectosigmoid junction): altered bowel habits with diarrhea and constipation; rectal bleeding and defecation pain. Rectovaginal Area: dysmenorrhea, dyspareunia, postcoital bleeding. Uterosacral ligaments: dyspareunia, dysmenorrhea, chronic pelvic pain.
Sliding Sign	Dynamic evaluation of vesicouterine and rectouterine pouch obliteration: assess bladder mobility relative to the uterus and bowel; assess cervical mobility relative to the rectum.	None or typical endometriosis symptoms: pelvic pain, dysmenorrhea, dyspareunia, infertility.
Soft Markers	Subjective findings in dynamic evaluation: ovarian mobility, localized tenderness.	Pelvic pain, dysmenorrhea, dyspareunia depending on location.

## Data Availability

No new data were created or analyzed in this study. Data sharing is not applicable to this article.

## References

[1] Zondervan K.T., Becker C.M., Koga K., Missmer S.A., Taylor R.N., Viganò P. (2018). Endometriosis. Nat. Rev. Dis. Primers.

[2] Zondervan K.T., Becker C.M., Missmer S.A. (2020). Endometriosis. N. Engl. J. Med..

[3] Burney R.O., Giudice L.C. (2012). Pathogenesis and pathophysiology of endometriosis. Fertil. Steril..

[4] Robert S., Schenken M.D. (2024). Endometriosis in Adults: Pathogenesis, Epidemiology, and Clinical Impact. https://www.uptodate.com/contents/endometriosis-in-adults-pathogenesis-epidemiology-and-clinical-impact.

[5] Amro B., Ramirez Aristondo M.E., Alsuwaidi S., Almaamari B., Hakim Z., Tahlak M., Wattiez A., Koninckx P.R. (2022). New Understanding of Diagnosis, Treatment and Prevention of Endometriosis. Int. J. Environ. Res. Public Health.

[6] National Guideline Alliance (UK) (2024). Endometriosis: Diagnosis and Management.

[7] Johnston J.L., Reid H., Hunter D. (2015). Diagnosing endometriosis in primary care: Clinical update. Br. J. Gen. Pract..

[8] Rolla E. (2019). Endometriosis: Advances and controversies in classification, pathogenesis, diagnosis, and treatment. F1000Res.

[9] Soliman A.M., Surrey E., Bonafede M., Nelson J.K., Castelli-Haley J. (2018). Real-World Evaluation of Direct and Indirect Economic Burden Among Endometriosis Patients in the United States. Adv. Ther..

[10] Nnoaham K.E., Hummelshoj L., Webster P., d’Hooghe T., de Cicco Nardone F., de Cicco Nardone C., Jenkinson C., Kennedy S.H., Zondervan K.T., World Endometriosis Research Foundation Global Study of Women’s Health consortium (2011). Impact of endometriosis on quality of life and work productivity: A multicenter study across ten countries. Fertil. Steril..

[11] Eisenberg V.H., Decter D.H., Chodick G., Shalev V., Weil C. (2022). Burden of Endometriosis: Infertility, Comorbidities, and Healthcare Resource Utilization. J. Clin. Med..

[12] Chapron C., Marcellin L., Borghese B., Santulli P. (2019). Rethinking mechanisms, diagnosis and management of endometriosis. Nat. Rev. Endocrinol..

[13] De Corte P., Klinghardt M., von Stockum S., Heinemann K. (2025). Time to Diagnose Endometriosis: Current Status, Challenges and Regional Characteristics—A Systematic Literature Review. BJOG Int. J. Obstet. Gynaecol..

[14] Villatoro A.R. (2024). Endometriosis. es. FMC—Formación Médica Continuada en Atención Primaria.

[15] Robert S., Schenken M.D. (2024). Endometriosis in Adults: Clinical Features, Evaluation, and Diagnosis. https://www.uptodate.com/contents/endometriosis-in-adults-clinical-features-evaluation-and-diagnosis.

[16] Sarria-Santamera A., Yemenkhan Y., Terzic M., Ortega M.A., Asunsolo Del Barco A. (2023). A Novel Classification of Endometriosis Based on Clusters of Comorbidities. Biomedicines.

[17] Medina-Perucha L., Pistillo A., Raventós B., Jacques-Aviñó C., Munrós-Feliu J., Martínez-Bueno C., Valls-Llobet C., Carmona F., López-Jiménez T., Pujolar-Díaz G. (2022). Endometriosis prevalence and incidence trends in a large population-based study in Catalonia (Spain) from 2009 to 2018. Womens Health.

[18] van der Zanden M., Teunissen D.A.M., van der Woord I.W., Braat D.D.M., Nelen W.L.D.M., Nap A.W. (2020). Barriers and facilitators to the timely diagnosis of endometriosis in primary care in the Netherlands. Fam. Pract..

[19] Melgaard A., Vestergaard C.H., Kesmodel U.S., Risør B.W., Forman A., Zondervan K., Bech B.H., Rytter D. (2023). Utilization of healthcare prior to endometriosis diagnosis: A Danish case-control study. Hum. Reprod..

[20] Edi R., Cheng T. (2022). Endometriosis: Evaluation and Treatment. Am. Fam. Physician.

[21] Saha R., Pettersson H.J., Svedberg P., Olovsson M., Bergqvist A., Marions L., Tornvall P., Kuja-Halkola R. (2015). Heritability of endometriosis. Fertil. Steril..

[22] Malinak L.R., Buttram V.C., Elias S., Simpson J.L. (1980). Heritage aspects of endometriosis. II. Clinical characteristics of familial endometriosis. Am. J. Obstet. Gynecol..

[23] Olšarová K., Mishra G.D. (2020). Early life factors for endometriosis: A systematic review. Hum. Reprod. Update.

[24] Shafrir A.L., Farland L.V., Shah D.K., Harris H.R., Kvaskoff M., Zondervan K., Missmer S.A. (2018). Risk for and consequences of endometriosis: A critical epidemiologic review. Best Pract. Res. Clin. Obstet. Gynaecol..

[25] Burton C., Iversen L., Bhattacharya S., Ayansina D., Saraswat L., Sleeman D. Pointers to Earlier Diagnosis of Endometriosis: A Nested Case-Control Study Using Primary Care Electronic Health Records. https://pubmed.ncbi.nlm.nih.gov/29109114/.

[26] de Kok L., Schers H., Boersen Z., Braat D., Teunissen D., Nap A. (2024). Towards reducing diagnostic delay in endometriosis in primary care: A qualitative study. BJGP Open.

[27] Macer M.L., Taylor H.S. (2012). Endometriosis and infertility: A review of the pathogenesis and treatment of endometriosis-associated infertility. Obstet. Gynecol. Clin. N. Am..

[28] Maqueda-Zamora G., Sierra-Santos L., Joleini-Joleini S., Krasnovska Zayets K., Maqueda-Zamora G., Sierra-Santos L., Joleini-Joleini S., Krasnovska Zayets K. (2024). No todo bulto umbilical es una hernia: Endometriosis primaria. Rev. Clínica Med. Fam..

[29] Berkley K.J., Rapkin A.J., Papka R.E. (2005). The pains of endometriosis. Science.

[30] Hare L., Roberts V., Hare N.P., Mughal F. (2023). Assessment and management of endometriosis in young people in primary care. Br. J. Gen. Pract..

[31] Dabi Y., Fauconnier A., Rousset-Jablonski C., Tavenet A., Pizzofferrato A.C., Deffieux X. (2024). Do women with suspected endometriosis benefit from pelvic examination to improve diagnostic and management strategy?. J. Gynecol. Obstet. Hum. Reprod..

[32] Mol B.W., Bayram N., Lijmer J.G., Wiegerinck M.A., Bongers M.Y., van der Veen F., Bossuyt P.M. (1998). The performance of CA-125 measurement in the detection of endometriosis: A meta-analysis. Fertil. Steril..

[33] Nisenblat V., Bossuyt P.M.M., Shaikh R., Farquhar C., Jordan V., Scheffers C.S., Mol B.W.J., Johnson N., Hull M.L. Blood Biomarkers for the Non-Invasive Diagnosis of Endometriosis. https://pubmed.ncbi.nlm.nih.gov/27132058/.

[34] Sociedad Espan ola de Ginecolog ıa y Obstetricia (2014). Introducci on Endometriosis (actualizado en febrero del 2013). Prog. Obstet. Ginecol..

[35] Agarwal S.K., Chapron C., Giudice L.C., Laufer M.R., Leyland N., Missmer S.A., Singh S.S., Taylor H.S. (2019). Clinical diagnosis of endometriosis: A call to action. Am. J. Obstet. Gynecol..

[36] Collins B.G., Ankola A., Gola S., McGillen K.L. (2019). Transvaginal US of Endometriosis: Looking Beyond the Endometrioma with a Dedicated Protocol. Radiographics.

[37] Nisenblat V., Bossuyt P.M.M., Farquhar C., Johnson N., Hull M.L. (2016). Imaging modalities for the non-invasive diagnosis of endometriosis. Cochrane Database Syst. Rev..

[38] Sayasneh A., Kaijser J., Preisler J., Smith A.A., Raslan F., Johnson S., Husicka R., Ferrara L., Stalder C., Ghaem-Maghami S. (2015). Accuracy of ultrasonography performed by examiners with varied training and experience in predicting specific pathology of adnexal masses. Ultrasound Obstet. Gynecol..

[39] Mick I., Freger S.M., van Keizerswaard J., Gholiof M., Leonardi M. (2024). Comprehensive endometriosis care: A modern multimodal approach for the treatment of pelvic pain and endometriosis. Ther. Adv. Reprod. Health.

[40] Robert S., Schenken M.D. (2024). Endometriosis: Medical Treatment of Pelvic Pain. https://www.uptodate.com/contents/endometriosis-medical-treatment-of-pelvic-pain?search=endometriosis&source=search_result&selectedTitle=2%7E150&usage_type=default&display_rank=2#H1186250471.

[41] Capezzuoli T., Rossi M., La Torre F., Vannuccini S., Petraglia F. (2022). Hormonal drugs for the treatment of endometriosis. Curr. Opin. Pharmacol..

[42] Becker C.M., Johnson N.P., As-Sanie S., Arjona Ferreira J.C., Abrao M.S., Wilk K., Imm S.J., Mathur V., Perry J.S., Wagman R.B. (2024). Two-year efficacy and safety of relugolix combination therapy in women with endometriosis-associated pain: SPIRIT open-label extension study. Hum. Reprod..

[43] As-Sanie S., Mackenzie S.C., Morrison L., Schrepf A., Zondervan K.T., Horne A.W., Missmer S.A. (2025). Endometriosis: A Review. JAMA.

[44] Karlsson J.V., Patel H., Premberg A. (2020). Experiences of health after dietary changes in endometriosis: A qualitative interview study. BMJ Open.

[45] Gao J., Liu H.Q., Wang Y., Shang Y.L., Hu F. (2019). Effects of psychological care in patients with endometriosis: A systematic review protocol. Medicine.

[46] Brooks T., Sharp R., Evans S., Baranoff J., Esterman A. (2021). Psychological Interventions for Women with Persistent Pelvic Pain: A Survey of Mental Health Clinicians. J. Multidiscip. Healthc..

[47] ADAEC Estatal: Adaec Estatal Asociación de Afectadas de Endometriosis. es. https://adaec.es/.

[48] Anastasiu C.V., Moga M.A., Elena Neculau A., Bălan A., Scârneciu I., Dragomir R.M., Dull A.M., Chicea L.M. (2020). Biomarkers for the Noninvasive Diagnosis of Endometriosis: State of the Art and Future Perspectives. Int. J. Mol. Sci..

[49] Simsa P., Mihalyi A., Kyama C.M., Mwenda J.M., Fülöp V., D’Hooghe T.M. (2007). Future of endometriosis research. Womens Health.

[50] Pant A., Moar K., KArora T., Maurya P.K. (2023). Biomarkers of endometriosis. Clin. Chim. Acta.

